# Use and Appreciation of a Web-Based, Tailored Intervention (E-health4Uth) Combined With Counseling to Promote Adolescents’ Health in Preventive Youth Health Care: Survey and Log-File Analysis

**DOI:** 10.2196/resprot.2855

**Published:** 2014-01-06

**Authors:** Rienke Bannink, Suzanne Broeren, Evelien Joosten-van Zwanenburg, Els van As, Petra van de Looij-Jansen, Hein Raat

**Affiliations:** ^1^Erasmus University Medical Center RotterdamDepartment of Public HealthRotterdamNetherlands; ^2^Regional Public Health & Youth Service South-Holland SouthDordrechtNetherlands; ^3^Consortium Rivas-CareynDepartment of Youth Health CareGorinchemNetherlands; ^4^Municipal Public Health Service Rotterdam areaRotterdamNetherlands

**Keywords:** adolescents, youth health care, Web-based tailoring, eHealth, Internet, counseling, health care evaluation, health promotion

## Abstract

**Background:**

Health promotion for adolescents is important in the prevention of mental health problems and health-risk behaviors. We implemented two interventions in a preventive youth health care setting. Adolescents in the E-health4Uth group received Web-based, tailored messages on their health behavior and well-being. Adolescents in the E-health4Uth and counseling group received the same tailored messages, but were subsequently referred to a school nurse for a consultation if they were at risk of mental health problems.

**Objective:**

This study evaluated the use and appreciation of these Web-based, tailored messages and additional consultation with a school nurse. Differences in use and appreciation according to demographics (ie, gender, level of education, and ethnicity) of the adolescents were also assessed.

**Methods:**

Two youth health care organizations participated in this study and conducted the interventions in 12 secondary schools. In total, 1702 adolescents participated; 533 in the E-health4Uth group, 554 in the E-health4Uth and counseling group, and 615 in the control group (ie, care as usual). Adolescents completed an evaluation questionnaire assessing the use and appreciation of the tailored messages immediately after receiving these messages and at a 4-month follow-up. After the consultation, adolescents and nurses completed an evaluation questionnaire on the use and appreciation of the consultation.

**Results:**

The majority of the adolescents (845/1034, 81.72%) indicated they had read the tailored messages. Most items on the use and appreciation of the tailored messages and the program were scored positive (overall satisfaction on a scale from 1, most-negative, to 10, most-positive: mean 6.70, SD 1.60). In general, adolescents in vocational training, girls, and adolescents of non-Dutch ethnicity, indicated they used the tailored messages more often and appreciated the content of the messages better than adolescents receiving preuniversity education, boys, and adolescents of Dutch ethnicity, respectively (all *P*<.05). In the E-health4Uth and counseling group, 18.6% (103/553) of the adolescents were referred to a nurse. Adolescents in vocational training and girls were more often referred to a nurse than adolescents receiving preuniversity education (*P*=.007) and boys (*P*=.03), respectively. Adolescents and nurses positively evaluated the consultation (overall satisfaction of adolescents: mean 8.07, SD 1.21). Adolescents in vocational training attended the consultation more often (*P*=.047) and considered the consultation a more valuable addition to the tailored messages than adolescents receiving preuniversity education (*P*=.034).

**Conclusions:**

The Web-based, tailored messages and additional consultation were used and appreciated positively by adolescents and nurses. The consultation seems a valuable addition to the tailored messages. However, the tailored messages might need further improvement since adolescents did not rate all evaluation items about these messages explicitly positive. As these interventions were already interweaved with the existing practice of the preventive youth health care, they are especially promising for future implementation.

**Trial Registration:**

Netherlands Trial Register Number (NTR): NTR3596; http://www.trialregister.nl/trialreg/admin/rctview.asp?TC=3596 (Archived by WebCite at http://www.webcitation.org/6LryL42zH).

## Introduction

Mental health problems often have their first manifestation during adolescence [1], and many health-risk behaviors, such as excessive alcohol consumption, cigarette smoking, drug use, and unsafe sex, are acquired during adolescence [2]. These mental health problems and health-risk behaviors often persist into adulthood, thereby affecting not only current health but also health later in life [3-8]. Therefore, adolescents are an important target group for health promotion.

Promoting good health and a healthy lifestyle is a task of the preventive youth health care [9]. The aim of preventive youth health care is to improve and protect the health, growth, and development of young people. In the Netherlands, all children and adolescents are invited for preventive periodic health examinations at set ages until the age of 13 years [10]. From the age of 5 years, these examinations often take place at school. The examinations focus on growth, development, health functioning, and behavior of infants, children, and adolescents. Given the rapid maturation in adolescence and the mental health problems and health-risk behaviors associated with this developmental period, it is desirable to implement an additional preventive health examination between ages 15 and 16 years [11,12]. 

Furthermore, with an increasing demand for adolescent health promotion by the government and preventive youth health care in the Netherlands [11,12], and the current financial strain on preventive health care, greater efficiency is required. Providing health information through the Internet (eHealth) can be beneficial for achieving this. For example, the Internet is very efficient for data sampling and offers the opportunity to give immediate computerized, tailored messages on health and health behavior [13,14]. Web-based, tailored messages eliminate (as far as possible) information that is not personally relevant [13,15-17] and are therefore more likely to be effective in changing behavior compared with nontailored messages [15]. Various studies have shown that Web-based tailoring is a promising technique to promote health behaviors of adolescents [18-23]. Additionally, it provides the opportunity to enhance the efficiency of face-to-face counseling by collecting information on adolescents’ health prior to the consultation, which could support the nurse during the consultation [24-28].

However, currently eHealth is not broadly applied in preventive youth health care, even though earlier research indicates that Web-based, tailored interventions can be combined with current daily practice of the preventive youth health care [28-30]. Therefore, we implemented two interventions in preventive youth health care using Web-based, tailored messages (E-health4Uth and E-health4Uth and counseling) [31]. These Web-based, tailored messages focused on topics related to health risk behaviors (eg, alcohol consumption, smoking) and well-being (eg, mental health status, suicidal thoughts). Both interventions used the same messages, which were developed for adolescents (aged 12-18 years) in an earlier study [32,33]. In the E-health4Uth and counseling group, adolescents who were at risk of mental health problems were also referred to a school nurse for a consultation. With adolescents’ knowledge, the nurses received information regarding adolescents’ health and health behaviors from the E-health4Uth tool, to facilitate communication during the consultation [28].

Evaluating use and appreciation of Web-based, tailored interventions and the consultation is important to guide improvement of interventions to increase intervention’s effectiveness [34]. Successful use and appreciation of an intervention are prerequisites for active information processing, which is necessary for achieving behavioral change [35,36]. Because demographic variables (gender, level of education, and ethnicity) have shown to influence the use of eHealth tools in general [37,38], research on the use and appreciation among specific demographic subgroups can provide insight into the usability of Web-based tailoring among such specific groups.

Taken together, the aim of this study was to evaluate the use and appreciation of the Web-based, tailored messages, and the use and appreciation of the subsequent consultation applied by the preventive youth health care in schools. Differences in use and appreciation according to demographics of the adolescents (by gender, level of education, and ethnicity) are explored.

## Methods

### Study Design

The study design is a three-armed cluster randomized controlled trial (RCT), with two intervention groups (E-health4Uth and E-health4Uth and counseling) and a control group (ie, care as usual). The interventions were applied by preventive youth health care in secondary schools. School classes were the unit of randomization, because randomization at the individual level (ie, the level of the adolescents) may lead to contamination of the control group [39]. For allocation of the school classes (clusters) to one of the study arms, a computer-generated list of random numbers was used. Randomization sequence was stratified with a 1:1:1 allocation using random block sizes of three. The computer-generated random number list was prepared by an investigator with no involvement in the trial. The random number list was applied by the researchers in the order schools committed to participate. This paper reports on the use and appreciation of the Web-based, tailored messages and counseling conducted in 2012 and 2013. Further details about the study design and the interventions are described in a design paper published elsewhere [31]. The Medical Ethical Committee of Erasmus Medical Center has declared that the Medical Research Involving Human Subjects Act (also known by its Dutch abbreviation WMO) does not apply to this research proposal. The Medical Ethical Committee had no objection against the execution of this research proposal (MEC-2012 - 337).

### Sample and Setting

Two youth health care organizations in the Dutch cities of Dordrecht and Zwijndrecht participated in this study and conducted the interventions in secondary schools. The youth health care organizations invited all 14 secondary schools in these cities to participate, of which 12 agreed with a total of 11 classes with third-grade students (2 schools) and 75 classes with fourth-grade students (10 schools). In the Netherlands, adolescents in the third- and fourth-grades of secondary school are on average 15-16 years of age. In secondary schools, distinction is made in the level of education adolescents are following. Lower levels of education are called “vocational training” and higher levels of education are called “preuniversity education”. Adolescents following vocational training and adolescents following preuniversity education both participated in this study.

**Figure 1:**
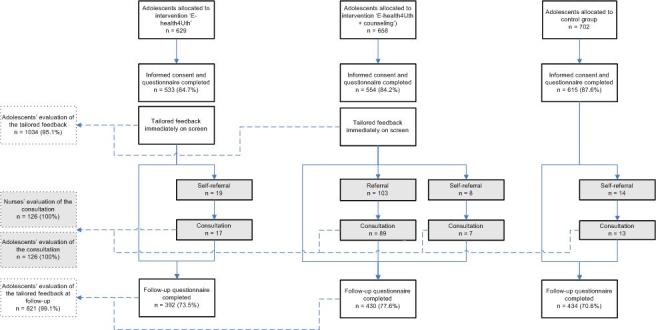
Flow chart of the adolescent's participation.

A few weeks prior to the start of the study, all adolescents and parents received information about the study. If parents did not want their child to participate, they could object to participation of their child. Adolescents were asked to provide written consent before they completed the baseline questionnaire. Of the 1989 eligible adolescents, 1702 (85.57%) adolescents participated; 533 in the E-health4Uth group, 554 in the E-health4Uth and counseling group, and 615 in the control group (Figure 1). The main registered reason for not participating was absence, mainly due to illness. Furthermore, 29 parents objected to their child’s participation, whereas 24 adolescents refused to participate. Of the 1087 adolescents who received the tailored messages (533 in the E-health4Uth group and 554 in the E-health4Uth and counseling group), 1034 (95.12%) completed the evaluation questionnaire at baseline.

At the 4-month follow-up, 3 schools did not succeed in scheduling the follow-up classroom assessments for all or several classes (missing data from 14 classes). At the remaining schools, 135 adolescents were absent at the follow-up. In total, 1256 adolescents participated at the 4-month follow-up (73.80%); 392 in the E-health4Uth group, 430 in the E-health4Uth and counseling group, and 434 in the control group. Of the 822 adolescents who participated at follow-up and received the tailored messages at baseline, 821 (99.9%) completed the evaluation questionnaire at follow-up.

All adolescents who attended the consultation with the nurse (n=126) completed the consultation evaluation questionnaire. Nurses also completed an evaluation questionnaire for every consultation (100%), but did not complete all questions.

### The E-health4Uth Intervention

**Figure 2:**
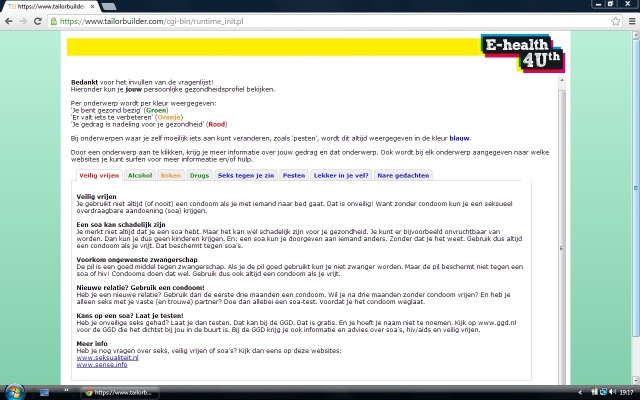
Screenshot of the computer-tailored messages. This is an example of a message (most left tab) that is presented to adolescents who have answered that they have had unsafe sex. The message is therefore displayed in red, indicating unhealthy behavior. By providing links to relevant websites, adolescents are encouraged to search for more information on the topic. The messages on the other topics are presented when clicking on the other (colored) tabs.

During one classroom session (+/- 45 minutes), adolescents completed a self-report questionnaire via the Internet to assess health-risk behavior and well-being on the following topics: alcohol consumption, drug use, smoking, sexual behavior, bullying, mental health status, suicidal thoughts, suicide attempts, and unpleasant sexual experiences (Multimedia Appendix 1). This questionnaire served as the basis to tailor the messages, but also as a baseline measure for the effect evaluation. For each topic, a score was computed that was compared with the Dutch health norms for adolescents [32,40]. Based on this score, a message was immediately presented on the screen, which reflected the person’s current behavior or well-being in relation to the Dutch health norm, and offered advice to change unhealthy behavior and/or to talk to a person the adolescent trusts (Figure 2). The messages were displayed in red, orange, or green, indicating unhealthy behavior, behavior just below the norm, or behavior according to the Dutch health norm, respectively. The topics on well-being were always displayed in blue. By providing links to relevant websites, adolescents were encouraged to read more information on the topics. These Web-based, tailored messages were specifically developed for adolescents (aged 12-18 years) in a previous study [32].

At the end of the program, adolescents were invited to follow the Facebook page of “E-health4Uth” to find additional information on the topics. Additionally, adolescents could check a box for a self-referral to the nurse or could send an email to the nurse. After 1 month, adolescents received a reminder of the tailored messages by email.

### The E-health4Uth and Counseling Intervention

During a classroom session, adolescents in the E-health4Uth and counseling group completed the questionnaire assessing health-risk behaviors and well-being. This was the same questionnaire as the one that was applied in the E-health4Uth-only group. They also received the tailored messages, and were invited to follow the Facebook page (see “The E-health4Uth intervention”). Adolescents could also check a box for a self-referral to the nurse or could send an email to the nurse.

Additionally, in this group adolescents at risk of mental health problems were invited for a consultation with the nurse. Adolescents were classified as at risk of mental health problems when: their score on the total problem scale of the Strengths and Difficulties Questionnaire (SDQ) was higher than 16, and/or their score on the SDQ emotional problems was higher than 5, and/or they reported having suicidal thoughts occasionally, more frequently, or did not want to answer this question, and/or they reported a suicide attempt last year or did not want to answer this question [31]. The consultation took place at school. The nurses received the results of the assessment for each referred adolescent prior to the consultation. During the consultation the nurses focused on specific risk areas and referred adolescents to other professionals if considered necessary.

### Control Group

Adolescents in the control group completed the same questionnaire assessing health-risk behaviors and well-being as adolescents in the intervention groups. The control group received care as usual (ie, adolescents could check a box for a self-referral with the nurse or could send an email to the nurse with any question or request for information or care). Due to the aim of this paper, this paper only uses this control group to assess the use and appreciation of the self-referred consultations (n=14).

### Measures

#### Evaluation of the Web-Based, Tailored Messages

Immediately after receiving the tailored messages and after 4 months, the adolescents were invited to complete an online evaluation questionnaire on the appreciation and use of the tailored messages. Effect outcome measures were also included, but these measures are described elsewhere [31], as they were not included in this study.

The use of the tailored messages was assessed with seven items. Immediately after adolescents received the tailored messages two items assessed the use of the messages: (1) reading the tailored messages (having read the messages completely/partly, or not at all), and (2) viewing websites to which reference was made (yes or no/not yet). Five items assessed the use of the tailored messages at the 4-month follow-up: (1) viewing the Facebook page of E-health4Uth (yes or no), (2) discussing messages with parents (yes or no), (3) discussing messages with peers (yes or no), (4) adhering to the advice, and (5) changing own behavior in a positive way. The last two items were scored on 5-point Likert scales ranging from 1 (totally disagree, most negative evaluation) to 5 (totally agree, most positive evaluation).

Appreciation of the tailored messages was assessed with 11 items immediately after the adolescents received the tailored messages. Six items assessed if the content of the tailored messages was credible, easy to understand, personally relevant, gave the adolescents insight into their own behavior, contained new information, and was attractive to read. A further two items assessed whether the adolescent learned a lot and appreciated to get information in this manner. Finally, three items measured overall satisfaction with the program, the ease of use of the program, and if the program was interesting. These items on the appreciation of the tailored messages and the program were all scored on a 5-point Likert scale ranging from 1 (totally disagree, most negative evaluation) to 5 (totally agree, most positive evaluation) with exception of the overall satisfaction with the program, which was scored on a scale from 1 (most negative evaluation) to 10 (most positive evaluation).

#### Evaluation of the Consultation

The use and appreciation of the consultation with the nurse was evaluated by computer log data and a paper-and-pencil questionnaire. That is, the number of adolescents that was referred or referred themselves to a nurse was measured objectively, based on computer log data. When adolescents attended the consultation, they were invited to complete a paper-and-pencil questionnaire about their appreciation of the consultation. The nurses noted whether or not the adolescents attended the consultation, and if the adolescent attended the consultation, they were invited to complete a written evaluation form regarding the consultation as well.

Various dimension of appreciation of the consultation were assessed among adolescents and nurses. One item measured the overall satisfaction with the consultation among adolescents on a scale from 1 (most-negative evaluation) to 10 (most-positive evaluation). Another two items evaluated the appreciation of being invited for consultation and whether the consultation was a valuable addition to the tailored messages among adolescents. These items were measured on 5-point Likert scales ranging from 1 (totally disagree, most negative evaluation) to 5 (totally agree, most positive evaluation). Two items addressed the nurse’s evaluations of whether the referral was legitimate and whether the information on the referred adolescents was helpful. These two items were also measured on a 5-point Likert scale ranging from 1 (not legitimate at all/very unhelpful) to 5 (completely legitimate/very helpful).

#### Demographics

Age (assessed by date of birth), gender, country of birth of the adolescent and both parents, and the level of education that the adolescents attended (ie, vocational or preuniversity education) were assessed in the evaluation questionnaire. Ethnicity was classified as Dutch or non-Dutch, in accordance with the definitions of Statistics Netherlands [40]. Adolescents with at least one parent born outside the Netherlands were classified as non-Dutch.

### Statistical Analysis

#### Overview

Descriptive statistics were used to describe both the study sample that received the tailored messages and the sample referred for consultation. Chi-square tests and independent samples *t* tests were conducted to test differences in demographic characteristics between both intervention groups. Descriptive statistics were also used to describe the use and appreciation of the tailored messages and the consultation. Chi-square tests (for dichotomous outcomes) and independent samples *t* tests or Mann-Whitney *U* tests (for ordinal outcomes) were conducted to test differences in use and appreciation according to: gender (boys versus girls), educational level (vocational versus preuniversity), and ethnicity (Dutch versus non-Dutch). Independent samples *t* tests were used for analysing data evaluating the tailored messages on 5- and 10-point scales. Because of the relative small sample size of the subsample receiving the consultation (n=126), data evaluating the consultation on 5- and 10-point scales were checked for normality. For ordinal variables that were nonnormally distributed, Mann-Whitney *U* tests were used.

Statistical analyses were performed using SPSS 20.0. Results were considered significant at *P*<.05.

#### Nonresponse Analysis

A comparison of adolescents participating at follow-up (n=822) with adolescents who were not participating at follow-up (*n*=265) did not indicate significant differences in terms of educational level (χ^2^_1_=1.92; *P*=.17) or gender (χ^2^_1_=0.64; *P=* .42). However, the group participating at follow-up was more often of Dutch ethnicity (χ^2^_1_=32.12; *P*<.001).

## Results

### Adolescents’ Characteristics

The average age of adolescents who received the tailored messages was 15.9 years (SD 0.72); 57.13% (621/1087) of the sample consisted of boys, 72.40% (787/1087) was of Dutch ethnicity, 52.53% (571/1087) attended vocational training, and 47.47% (516/1087) preuniversity education (Table 1). Although adolescents in the E-health4Uth group were significantly younger than adolescents in the E-health4Uth and counseling group, the actual mean age difference was very small (mean 15.9, SD 0.73 vs mean 16.0, SD 0.70, *P*=.02, respectively).

**Table 1:** General characteristics of the study population, and by intervention group (N=1087).

	Total N=1087	E-health4Uth n=533	E-health4Uth and counseling n=554	*P* value
Age in years, mean (SD)	15.9 (0.72)	15.9 (0.73)	16.0 (0.70)	.02^a^
Boys, n (%)	621 (57.13)	294 (55.16)	327 (59.03)	.20^b^
Ethnicity, Dutch, n (%)	787 (72.40)	395 (74.11)	392 (70.76)	.22^b^
Educational level, vocational training, n (%)	571 (52.53)	265 (49.72)	306 (55.23)	.07^b^

^a^Independent samples *t* tests

^b^χ^2^ tests

### Adolescents’ Use of the Web-Based, Tailored Messages

The results regarding the use of the tailored messages (E-health4Uth) are shown in Tables 2 and 3. During the school session, 81.72% (845/1034) of the adolescents read the messages, whereas 4.5% (38/841) of these adolescents also viewed the websites to which reference was made in these messages. After 4 months, 3.6% (29/814) of the adolescents had viewed the Facebook page of E-health4Uth. Of the adolescents who reported at follow-up that they read the messages, 18.4% (105/572) had discussed these messages with their parents and 24.0% (137/572) with their peers, 41.1% (235/572) reported that they could adhere to advice, and 21.5% (123/572) indicated that the messages changed their behavior in a positive way.

Adolescents receiving preuniversity education read the tailored messages more often than adolescents in vocational training (*P*<.001), whereas adolescents in vocational training more often viewed the websites to which they were referred (*P*<.001), more often indicated that they could adhere to advice (*P*<.001), and had changed their behavior accordingly in a positive way (*P*<.001). Adolescents of Dutch ethnicity and girls discussed the messages more often with their peers than adolescents of non-Dutch ethnicity (*P*<.02) and boys (*P*<.04), whereas adolescents of non-Dutch ethnicity more often indicated that they could adhere to advice (*P*<.005).

### Adolescents’ Appreciation of the Web-Based, Tailored Messages

Of the adolescents who had read the messages, a large majority (703/843, 83.4%; mean 4.02, SD 0.83) was positive about the ease to understand the messages (Table 2). More than half of the adolescents found the messages credible (510/843, 60.5%; mean 3.59, SD 0.96) and the program easy to use (557/843, 66.1%; mean 3.68, SD 0.93). On six other items, the adolescents evaluated the messages and the program about neutral (score 3 reflects not negative/not positive): personal relevance (mean 3.21, SD 1.06), appreciated getting information in this manner (mean 3.15, SD 1.02), gave insight into own behavior (mean 2.83, SD 1.11), attractive to read (mean 2.89, SD 1.05), learned a lot (mean 2.80, SD 1.07), and program was interesting (mean 2.96, SD 1.06). In general, adolescents evaluated the messages on containing new information as slightly negative (mean 2.44, SD 1.14), indicating that at least a part of the information in the messages was not new to the adolescents. Furthermore, adolescent’s mean rating of the E-health4Uth program was positive, namely a 6.70 (SD 1.60) on a scale from 1 (most-negative evaluation) to 10 (most-positive evaluation).

When considering subgroups, adolescents receiving preuniversity education considered the messages easier to understand (*P*=.03) and the program easier to use (*P*=.01) than adolescents in vocational training (Table 3). Adolescents in vocational training appreciated the messages better than adolescents receiving preuniversity education on three items; they rated the messages as containing more novel information (*P*<.001), providing them more insight into their own behavior (*P*<.001), and more instructive (*P*<.001). Adolescents of non-Dutch ethnicity also appreciated these three items better (ie, contained new information, *P*=.002; gained insight into own behavior, *P*=.02; and learned a lot, *P*=.002), and they rated the program as more interesting than adolescents of Dutch ethnicity (*P*=.004)*.* Furthermore, girls appreciated the messages and the program better than boys; girls found the messages more credible (*P*=.03) and easier to understand (*P*=.002). Furthermore, they were more satisfied with the program (*P*=.005), found the program easier to use (*P*=.004), and more interesting (*P*=.006) than boys.

**Table 2:** Adolescents’ use and appreciation of the tailored messages and the E-health4Uth program for the study.

		Total sample		Educational level		
				Vocational	Preuniversity	*P* value
		n (%)	Mean (SD)	n (%) or mean (SD)	n (%) or mean (SD)	
**Use tailored messages**							
	Read during school session	845/1034 (81.72)		407/533 (76.36)	438/501 (87.43)	<.001^a^
	Viewed websites to which reference was made (when read messages)	38/841 (4.52)		31/403 (7.69)	7/438 (1.60)	<.001^a^
	Viewed Facebook page of E-health4Uth^b^	29/814 (3.56)		18/415 (4.34)	11/399 (2.76)	.22
	Discussed with parents^b^	105/572 (18.36)		57/270 (21.11)	48/302 (15.89)	.11
	Discussed with peers^b^	137/572 (23.95)		59/270 (21.85)	78/302 (25.83)	.27
	Could adhere to advice^b,c^	235/572 (41.08)^e^	3.24 (1.14)	3.47 (1.13)	3.04 (1.11)	<.001^a^
	Changed own behavior in a positive way^b,c^	123/572 (21.50)^e^	2.69 (1.19)	2.89 (1.23)	2.52 (1.12)	<.001^a^
**Appreciation content-tailored messages** ^c^						
	Credible	510/843 (60.50)^e^	3.59 (0.96)	3.60 (0.98)	3.58 (0.94)	.70
	Easy to understand	703/843 (83.39)^e^	4.02 (0.83)	3.93 (0.92)	4.10 (0.74)	.003^a^
	Personally relevant	356/843 (42.23)^e^	3.21 (1.06)	3.27 (1.03)	3.17 (1.08)	.17
	Gave insight into own behavior	234/843 (28.83)^e^	2.83 (1.11)	3.00 (1.12)	2.67 (1.08)	<.001^a^
	Contained new information	162/843 (19.22)^e^	2.44 (1.14)	2.71 (1.18)	2.20 (1.05)	<.001^a^
	Attractive to read	243/843 (28.83)^e^	2.89 (1.05)	2.94 (1.09)	2.85 (1.01)	.26
	Learned a lot	212/843 (25.15)^e^	2.80 (1.07)	2.95 (1.11)	2.66 (1.01)	<.001^a^
	Appreciated getting information in this manner	332/843 (39.38)^e^	3.15 (1.02)	3.19 (1.05)	3.12 (0.99)	.36
**Appreciation E-health4Uth program**							
	Overall satisfaction^d^		6.70 (1.60)	6.77 (1.81)	6.64 (1.39)	.26
	Easy to use^c^	557/843 (66.07)^e^	3.68 (0.93)	3.60 (0.95)	3.76 (0.91)	.01^a^
	Interesting^c^	261/843 (30.96)^e^	2.96 (1.06)	3.03 (1.09)	2.89 (1.03)	.06

^a^Indicate significant *P* values.

^b^Measured at follow-up.

^c^Scores on a 5-point Likert scale ranging from 1 (totally disagree) to 5 (totally agree).

^d^Scores on a 10-point Likert scale ranging from 1 (most-negative evaluation) to 10 (most-positive evaluation).

^e^Percentages of adolescents who scored a 4 agree or 5 totally agree on the 5-point Likert scale.

**Table 3:** Adolescents’ use and appreciation of the tailored messages and the E-health4Uth program by gender and ethnicity.

		Gender			Ethnicity		
		Boys	Girls	*P* value	Dutch	Non-Dutch	*P* value
		n (%) or mean (SD)	n (%) or mean (SD)		n (%) or mean (SD)	n (%) or mean (SD)	
**Use tailored messages**							
	Read during school session	473/590 (80.17)	372/444 (83.78)	.14	624/755 (82.65)	221/279 (79.21)	.20
	Viewed websites to which reference was made (when read messages)	25/467 (5.35)	13/374 (3.48)	.19	23/621 (3.70)	15/220 (6.82)	.06
	Viewed Facebook page of E-health4Uth^a^	16/458 (3.49)	13/356 (3.65)	.90	22/625 (3.52)	7/189 (3.70)	.90
	Discussed with parents^a^	45/290 (15.52)	60/282 (21.28)	.08	83/449 (18.49)	22/123 (17.89)	.88
	Discussed with peers^a^	59/290 (20.34)	78/282 (27.66)	.04^d^	117/449 (26.06)	20/123 (16.26)	.02^d^
	Could adhere to advice^a,b^	3.21 (1.19)	3.28 (1.09)	.46	3.17 (1.12)	3.50 (1.16)	.005^d^
	Changed own behavior in a positive way^a,b^	2.69 (1.22)	2.70 (1.15)	.98	2.71 (1.16)	2.65 (1.28)	.65
**Appreciated content-tailored messages** ^b^ **, mean (SD)**							
	Credible	3.53 (1.00)	3.67 (0.89)	.03^d^	3.57 (0.92)	3.65 (1.05)	.30
	Easy to understand	3.94 (0.91)	4.11 (0.71)	.002^d^	4.01 (0.80)	4.02 (0.93)	.91
	Personally relevant	3.21 (1.08)	3.22 (1.03)	.96	3.19 (1.03)	3.28 (1.23)	.27
	Gave insight into own behavior	2.79 (1.15)	2.88 (1.06)	.21	2.78 (1.09)	2.98 (1.16)	.02^d^
	Contained new information	2.41 (1.16)	2.48 (1.11)	.42	2.37 (1.11)	2.64 (1.21)	.002^d^
	Attractive to read	2.84 (1.08)	2.96 (1.02)	.10	2.87 (1.02)	2.96 (1.14)	.25
	Learned a lot	2.76 (1.12)	2.85 (1.00)	.22	2.73 (1.02)	3.00 (1.18)	.002^d^
	Appreciated getting information in this manner	3.12 (1.05)	3.20 (0.98)	.27	3.13 (1.01)	3.20 (1.05)	.42
**Appreciation E-health4Uth program, mean (SD)**							
	Overall satisfaction^c^	6.57 (1.75)	6.87 (1.39)	.005^d^	6.72 (1.54)	6.65 (1.78)	.56
	Easy to use^b^	3.60 (0.98)	3.79 (0.86)	.004^d^	3.72 (0.90)	3.58 (1.02)	.08
	Interesting^b^	2.87 (1.07)	3.07 (1.04)	.006^d^	2.90 (1.04)	3.13 (1.11)	.004^d^


^a^Measured at follow-up.

^b^Scores on a 5-point Likert scale ranging from 1 (totally disagree) to 5 (totally agree).

^c^Scores on a 10-point Likert scale ranging from 1 (most-negative evaluation) to 10 (most-positive evaluation).

^d^Indicate significant *P* values.

### Adolescents’ Use of the Consultation

The results regarding the use of the consultation with the nurse are shown in Tables 4 and 5. Of the 554 adolescents in the E-health4Uth and counseling group, 103 (18.6%) adolescents were referred to a nurse. Adolescents were most often referred based on a high score (>16) on the total problem scale of the SDQ (12.1% of the 18.6%, ie, 65.0%; Table 4). Adolescents in the two intervention groups and the control group could also check a box for a self-referral; 44 of the 1702 adolescents checked the box for a self-referral (2.6%). Three of these 44 adolescents were in the E-health4Uth and counselling group and at risk of mental health problems, and therefore already referred to a nurse.

One hundred twenty-six of 144 adolescents who were referred or self-referred attended the consultation (87.5%). The average age of adolescents who presented for the consultations was 16 years (SD 0.73); 50.7% of this sample consisted of boys, 61.4% was of Dutch ethnicity, and 66.4% attended vocational training.

Adolescents in vocational training were more often referred to a nurse because of a high total problem score on the SDQ than adolescents receiving preuniversity education (*P=*.035), whereas girls and adolescents of Dutch ethnicity were more often referred to a nurse because of a high score on the SDQ emotional problems subscale than boys (*P*<.001) and adolescents of non-Dutch ethnicity (*P*=.039). Adolescents of non-Dutch ethnicity were more often referred to a nurse because they did not want to answer the question about suicidal thoughts (*P*=.002) and/or suicide attempts (*P*=.001) than adolescents of Dutch ethnicity. Adolescents of non-Dutch ethnicity more often asked for a referral than adolescents of Dutch ethnicity (*P*<.001), whereas adolescents in vocational training attended the consultation more often than adolescents receiving preuniversity education (*P*=.047).

**Table 4:** Description of adolescents’ use and appreciation of the consultation for the study sample and by educational level.

		Total sample		Educational level		
				Vocational	Preuniversity	*P* value
		n (%)	Mean (SD)	n (%), or mean (SD)	n (%), or mean (SD)	
**Use of consultation**						
	Referred to a nurse	103/553 (18.62)		69/305 (22.62)	34/248 (13.71)	.007^f^
	Total SDQ score>16	67/553 (12.11)		45/305 (14.75)	22/248 (8.87)	.035^f^
	SDQ subscale emotional problems>5	40/553 (7.23)		24/305 (7.87)	16/248 (6.45)	.52
	Suicidal thoughts ‘occasionally’ or more often	22/553 (3.98)		12/305 (3.93)	10/248 (4.03)	.95
	Did not want to answer question about suicidal thoughts	26/553 (4.70)		19/305 (6.23)	7/248 (2.82)	.06
	Suicide attempt last year	4/553 (0.72)		3/305 (0.98)	1/248 (0.40)	.42
	Did not want to answer question about suicidal attempt last year	20/553 (3.61)		14/305 (4.59)	6/248 (2.42)	.17
	Asked for a referral	44/1702 (2.59)^a^		27/914 (2.95)	17/788 (2.16)	.30
	Attending consultation	126/144 (87.50)		86/94 (91.49)	40/50 (80.00)	.047^f^
**Adolescent, Appreciation of consultation**						
	Overall satisfaction^b^		8.07 (1.21)	8.07 (1.00)	8.08 (1.58)	.42^e^
	Appreciated to be invited^c^	83/126 (65.9)^d^	3.70 (1.10)	3.81 (1.05)	3.45 (1.18)	.09^e^
	Valuable addition to the tailored messages^c^	81/126 (64.3)^d^	3.85 (1.04)	3.99 (0.98)	3.55 (1.11)	.03^e,f^
**Nurse, Appreciation of consultation**						
	Referral was legitimate^c^	63/89 (70.8)^d^	3.53 (1.00)	3.60 (0.99)	3.35 (1.02)	.21^e^
	Self-referral was legitimate^c^	21/37 (56.8)^d^	3.30 (1.15)	3.17 (1.19)	3.50 (1.09)	.45^e^
	Information of the adolescent was helpful^c^	88/110 (80.0)^d^	3.83 (0.86)	3.75 (0.92)	3.97 (0.71)	.21^e^

^a^Three of the 44 adolescents who asked for a referral were in the E-health4Uth + counselling group and at risk of mental health problems, and therefore also referred to a nurse.

^b^Scores on a 10-point Likert scale ranging from 1 (most-negative evaluation) to 10 (most-positive evaluation).

^c^Scores on a 5-point Likert scale ranging from 1 (most-negative evaluation) to 5 (most-positive evaluation).

^d^Percentages of adolescents or nurses who scored a 4 agree/legitimate/helpful or 5 totally agree/completely legitimate/very helpful on the 5-point Likert scale.

^e^Mann-Whitney *U* test.

^f^Indicate significant *P* values.

**Table 5:** Description of adolescents’ use and appreciation of the consultation by gender and ethnicity.

		Gender				Ethnicity		
		Boys		Girls	*P* value	Dutch	Non-Dutch	*P* value
		n (%), or mean SD		n (%), or mean SD		n (%), or mean SD	n (%), or mean SD	
**Use of consultation, n (%)**									
	Referred to a nurse	51/326 (15.64)		52/227 (22.91)	.03^d^	69/391 (17.65)	34/162 (20.99)	.36
	Total SDQ score>16	37/326 (11.35)		30/227 (13.22)	.51	45/391 (11.51)	22/162 (13.58)	.50
	SDQ subscale emotional problems>5	11/326 (3.37)		29/227 (12.78)	<.001^d^	34/391 (8.70)	6/162 (3.70)	.039^d^
	Suicidal thoughts ‘occasionally’ or more often	13/326 (3.99)		9/227 (3.96)	.99	17/391 (4.35)	5/162 (3.09)	.49
	Did not want to answer question about suicidal thoughts	14/326 (4.29)		12/227 (5.29)	.59	11/391 (2.81)	15/162 (9.26)	.001^d^
	Suicide attempt last year	2/326 (0.61)		2/227 (0.88)	.72	4/391 (1.00)	0/162 (0.00)	.20
	Did not want to answer question about suicidal attempt last year	11/326 (3.37)		9/227 (3.96)	.71	8/391 (2.05)	12/162 (7.41)	.002^d^
	Asked for a referral	24/906 (2.65)		20/796 (2.51)	.86	20/1207 (1.66)	24/495 (4.85)	<.001^d^
	Attending consultation	62/73 (84.93)		64/71 (90.14)	.35	74/88 (84.09)	52/56 (92.86)	.12
**Adolescent, appreciation of consultation, mean (SD)**									
	Overall satisfaction^a^	8.20 (0.98)		7.95 (1.39)	.47^c^	8.10 (0.97)	8.04 (1.50)	.85^c^
	Appreciated to be invited^b^	3.69 (0.97)		3.70 (1.22)	.55^c^	3.59 (1.03)	3.85 (1.18)	.11^c^
	Valuable addition to the tailored messages^b^	3.92 (1.00)		3.78 (1.08)	.50^c^	3.70 (1.14)	4.06 (0.83)	.13^c^
**Nurse, appreciation of consultation, mean (SD)**									
	Referral was legitimate^b^	3.53 (0.98)		3.52 (1.03)	.90^c^	3.66 (0.88)	3.27 (1.17)	.16^c^
	Self-referral was legitimate^b^	3.00 (1.20)		3.61 (1.04)	.13^c^	3.07 (1.16)	3.45 (1.14)	.33^c^
	Information of the adolescent was helpful^b^	3.73 (1.01)		3.90 (0.72)	.47^c^	3.98 (0.62)	3.59 (1.09)	.06^c^

^a^Scores on a 10-point Likert scale ranging from 1 (most-negative evaluation) to 10 (most-positive evaluation).

^b^Scores on a 5-point Likert scale ranging from 1 (most-negative evaluation) to 5 (most-positive evaluation).

^c^Mann-Whitney *U* test.

^*d*^Indicate significant *P* values.

### Adolescents’ and Nurses’ Appreciation of the Consultation

Adolescents appreciated being invited for the consultation (mean 3.70, SD 1.10), found the consultation a valuable addition to the tailored messages (mean 3.86, SD 1.03), and they gave the consultation a positive mean rating of 8.07 on a 10-point scale (SD 1.21). Adolescents in vocational training considered the consultation a more valuable addition to the Web-based, tailored messages than adolescents receiving preuniversity education (*P*=.034).

After the consultation, nurses evaluated most referrals for the adolescents at risk of mental health problems (63/89, 70.8%; mean 3.53, SD 1.00) and for adolescents that self-referred (21/37, 56.8%; mean 3.30, SD 1.15) as legitimate. In most cases (88/110, 80.0; mean 3.83, SD 0.86), the nurses also rated the information they received about the adolescents prior to the consultation as helpful.

## Discussion

### Principal Results

In the present study, we evaluated the use and appreciation of two interventions (E-health4Uth and E-health4Uth and counseling) applied by preventive youth health care in secondary schools. Results showed that most adolescents had read the tailored messages and evaluated the use and appreciation of the tailored messages and the E-health4Uth program overall as positive. In general, adolescents in vocational training, girls, and adolescents of non-Dutch ethnicity used the Web-based, tailored messages more and appreciated them better than adolescents receiving preuniversity education, boys, and adolescents of Dutch ethnicity, respectively. Adolescents in vocational training and girls were more often referred to a nurse than adolescents receiving preuniversity education and boys, respectively. Adolescents of Dutch and non-Dutch ethnicity were as often referred to a nurse, but for different reasons. Adolescents of Dutch ethnicity were more often referred because of a high score on the SDQ emotional problems subscale, whereas adolescents of non-Dutch ethnicity were more often referred because they did not want to answer questions about suicidal thoughts and suicide attempts. Adolescents of non-Dutch ethnicity asked for a referral more often than adolescents of Dutch ethnicity, whereas adolescents in vocational training attended the consultation more often than adolescents receiving preuniversity education. The adolescents who attended the consultation evaluated the consultation positively, just as the nurses did. Adolescents, especially those in vocational training, considered the consultation a valuable addition to the Web-based, tailored messages.

### Interpretation

This study indicates that Web-based tailoring is useful in a preventive-care setting to provide adolescents with information about their lifestyle behaviors and well-being. Other studies in which tailored messages were used among adolescents have shown comparable ratings of use and appreciation of tailored messages about health and healthy behavior [22,41,42]. Adolescents in the E-MOVO (Electronic Monitor and Health Education) study, in which similar tailored messages were used, appreciated the messages slightly more than adolescents in our study with regard to credibility, personal relevance, giving insight into own behavior, and ease of understanding [43]. However, in the E-MOVO study the response rate of the evaluation questionnaire was very low (ie, only 3%). Therefore, perhaps only the highly-motivated adolescents completed the evaluation questionnaire, which could have resulted in a more positive rating.

Results of earlier studies [28,41] and this study show that a vast majority of the adolescents had read the messages. This indicates that adolescents are interested in receiving feedback on lifestyle behaviors and well-being when communicated through the Internet. Furthermore, Web-based tailoring seems an appropriate way to adjust messages to adolescents’ needs of information on their lifestyle behaviors and well-being in a preventive-care setting. It must be noted though that the percentage of adolescents that clicked on a link or viewed the Facebook page to obtain additional information on the various topics in the intervention was relatively low. This could be due to the messages already containing enough information, the adolescents not wanting to obtain more information, or adolescents not wanting to be continually sent “off-site” from the intervention page to view information [44]. Beside these explanations, visiting Facebook is often not allowed at schools, and therefore maybe not preferable to use in this context.

Furthermore, approximately 20% of the adolescents indicated they had discussed the messages with their parents or peers. In a study of Ezendam et al [41], which used Web-based, tailored messages on dietary and physical activity, 40% of the adolescents indicated that they discussed the messages with their parents or peers. Although in our study, fewer adolescents discussed the messages with their parents or peers, this still may be an indication that adolescents, to some extent, actively process the information, a prerequisite for behavior change [35,36]. Moreover, the rationale behind using tailored messages is that the information is personally relevant, new, giving insight into own behavior, and is interesting, which results in greater attention and more thoughtful consideration of the information [13,45]. In our study, these items regarding personal relevance, giving insight into own behavior, and finding it interesting were evaluated as neutral and at least part of the information in the messages was not new to the adolescents. Therefore, the tailored messages in this study possibly need further improvement, which may result in the messages becoming even more effective. The current messages could be further tailored using, for example, demographics, personal cognitive factors (eg, manner in which health risks are perceived by the individual), social factors (eg, susceptibility to social pressure from peers), or self-efficacy of the individual (eg, judgement of capability to change unhealthy behavior) [46,47].

The differences in use and appreciation of the tailored messages according to demographics of the adolescents found in our study also support the need to further tailor the messages to the individual adolescents’ needs. That is, in line with the research of Ezendam et al [41], in our study, adolescents receiving preuniversity education perceived the messages as easier to understand than adolescents in vocational training. An explanation for this finding, which is supported by research of Ezendam et al [41] and our results on the novelty of the information, may be that the information was already familiar to adolescents receiving preuniversity education. Therefore, we suggest that messages should be tailored to educational level. This is important as lesser-educated adolescents tend to have a less healthy lifestyle and they rate their well-being as lower compared with higher-educated people [2,41,48]. However, effects for other demographics were also found. Additional analyses (data not shown) showed that the various demographics (ie, level of education, gender, and ethnicity) had an effect on the use and appreciation of the tailored messages independent of each other, indicating it is important to use multiple characteristics to best tailor these messages.

Although the tailored messages have the potential to reach large groups of adolescents in a very cost-effective manner [13], the consultation with the nurse was rated more positively. This was supported by additional analyses with the subsample of adolescents who evaluated the tailored messages and received and evaluated the consultation. These analyses showed similar results on the use and appreciation of the tailored messages for the subsample of adolescents compared with the whole group of adolescents (data not shown). The more positive evaluation of the consultation in comparison with the tailored messages could have been due to the interaction between the nurse and adolescent during the consultation, as previous research has shown that interaction in health communication could improve patient satisfaction [49,50]. Furthermore, the collected information on adolescents’ health prior to the consultation could have supported the nurse during the consultation to better tailor the provided information to the adolescent’s needs. Sciamanna et al [27] have shown that discussing previously collected information with a patient during a consultation could improve patient satisfaction as well. Therefore, the consultation seems a valuable addition to the Web-based, tailored messages for adolescents at risk of mental health problems and for adolescents wanting a referral to the nurse.

Finally, as expected, approximately one-fifth of the adolescents met the inclusion criteria for an appointment with the nurse [31]. In line with previous studies [2,51], adolescents in vocational training were more often at risk of mental health problems than adolescents receiving preuniversity education, whereas girls and adolescents of Dutch ethnicity were more often at risk of emotional problems than boys and adolescents of non-Dutch ethnicity. Adolescents of non-Dutch ethnicity more often did not want to answer the questions about suicidal thoughts and suicide attempt. It is possible that the stigma associated with suicidal thoughts and suicide attempts among some cultural groups may have contributed to not wanting to answer the questions regarding these issues [52,53]. Adolescents of non-Dutch ethnicity more often asked for a referral than adolescents of Dutch ethnicity, which is in line with, for example, the more frequent use of the general practitioner by ethnic minorities in the Netherlands [54].

In this study, as well as in another study in a similar setting [28], most adolescents attended the consultation, and in most cases nurses evaluated the referral as legitimate and the information they received on the adolescent prior to the consultation as helpful. This may indicate that the criteria we used to select adolescents at risk of mental health problems were suitable and selected adolescents were willing to attend the consultation with the nurse. Furthermore, the information regarding adolescents’ health and health behavior from the E-health4Uth tool to facilitate communication during consultation seemed appropriate.

### Strengths and Limitations

The response rate on the evaluation questionnaires was relatively high and our study population resembles the average Dutch adolescent population in secondary schools in gender, ethnicity, and educational level [55]. However, this study was only conducted among Dutch adolescents of ages 15-16 years in a preventive-care setting and therefore generalization to other countries, age groups, and settings should be done with caution. Because adolescents’ response was not anonymous, but confidential due to the necessity to match the follow-up data and to provide the nurse with information about the adolescents who were invited for the consultation, this could have had an effect on the social desirability of the adolescent’s responses. However, a previous study showed that anonymous and confidential collection of data revealed similar results on adolescents’ self-report measures of various (psychological) health indicators [56].

A strength of this study is the focus on multiple behaviors, which is also becoming an increasingly popular strategy in research on the effectiveness of Web-based, tailored messages [57-60]. However, due to this focus on multiple behaviors, adolescents received a lot of information and it is conceivable that adolescents became overwhelmed due to the amount of information and may have read the information less carefully. Future studies might therefore consider reducing the number of topics or offering the multiple tailored messages consecutively at different points in time [60]. Moreover, the current messages could be further tailored by using demographics, personal cognitive factors, social factors, or self-efficacy of the individual to show healthy behavior.

A strength of the interventions is the adaptability to other settings. For example, the tailored messages could be embedded in the school’s health promotion curriculum, and offered at relevant moments in the curriculum. But it is also possible that the assessment takes place at home and the subsequent consultation at the preventive health care organization. However, future research is required to investigate the application of the interventions in other settings. Finally, it is possible that some adolescents have mainly read the messages because they had to attend their class anyway. Nevertheless, our study showed that the adolescents rated the messages overall as positive, indicating that regardless of their motivation to participate they appreciated the content of the messages.

### Conclusions

The Web-based, tailored messages and additional consultation were used and appreciated positively by adolescents and nurses. The consultation seems a valuable addition to the tailored messages for adolescents at risk of mental health problems and for adolescents wanting a referral to the nurse. However, the tailored messages may need further improvement, since adolescents did not rate all the evaluation items on the messages as positive. As these interventions were already interweaved with the existing practice of the preventive youth health care, they are especially promising for future implementation. Furthermore, algorithms generating tailored information can be easily extended using more characteristics of the adolescent to tailor the messages, and wide-scale distribution can be arranged at relatively low cost. Future research is necessary to investigate the possible effects of the Web-based, tailored messages and the consultation with the nurse on the well-being and health behaviors of adolescents.

## Multimedia Appendix 1

Topics of the eHealth modules.

## Abbreviations

E-MOVO: Electronic Monitor and Health Education
RCT: randomized controlled trial
SDQ: Strengths and Difficulties Questionnaire
WMO: Wet Medisch wetenschappelijk Onderzoek met mensen
