# Supplementary material for: Use and Appreciation of a Web-Based, Tailored Intervention (E-health4Uth) Combined With Counseling to Promote Adolescents’ Health in Preventive Youth Health Care: Survey and Log-File Analysis
Source: JMIR Res Protoc. 2014 Jan 6;3(1):e3. doi: 10.2196/resprot.2855 (PMC3906651; doi:10.2196/resprot.2855)
Supplement: Supplementary file 1 [file resprot_v3i1e61_app1.pdf]

Appendix 1: Topics of the E-health modules

Behavior and well-being	Items
Alcohol consumption	How often and how much the adolescent drinks alcohol (9 items)
Drugs use	How often the adolescents has used different types of drugs (17 items)
Smoking	How often the adolescent smokes (2 items)
Sexual behavior	How often the adolescent uses condoms during sexual intercourse (2 items)
Bullying	How often the adolescent is bullied at school, somewhere else, or on the internet (3 items)
Mental health status	Strength and Difficulties Questionnaire (SDQ) (25 items) with a total score range 0-40
Suicidal thoughts	If the adolescent has had suicidal thoughts last year (1 item)
Suicidal attempts	If the adolescent made a suicidal attempt last year (1 item)
Unpleasant sexual experience	If the adolescent has ever had an unpleasant sexual experience (1 item)
